# Scleroderma Renal Crisis With Thrombotic Microangiopathy Treated With Eculizumab

**DOI:** 10.7759/cureus.31977

**Published:** 2022-11-28

**Authors:** Ludovic Saba, Joseph Kassab, Vivek Mehta, Mohammed Bari

**Affiliations:** 1 Department of Internal Medicine, Saint Joseph University of Beirut, Beirut, LBN; 2 Department of Rheumatology, Alaska Native Tribal Health Consortium, Anchorage, USA; 3 Department of Rheumatology, Houston Methodist Sugar Land Hospital, Sugar Land, USA

**Keywords:** autoimmune diseases, monoclonal antibody, immunology, hypertension, renal failure, scleroderma

## Abstract

We herein report the unusual case of a 52-year-old female with systemic scleroderma who was admitted to the emergency department (ED) with renal dysfunction and hypertension. Following a decline in hemoglobin (Hb) and platelet (Plt) count, the diagnosis of scleroderma renal crisis (SRC) with associated microangiopathic hemolytic anemia was made. Renal replacement therapy using hemodialysis was required. Systemic scleroderma is a chronic autoimmune multisystem vasculopathy affecting several vessel beds, including distal extremities, kidneys, and lungs. Microangiopathic hemolytic anemia occurs in almost half of patients who develop scleroderma renal crisis. This association is thought to be related to the activation of the complement system via the classical pathway. Based on that, we administered a C5 blocker (eculizumab) to our patient and reported an unprecedented positive outcome.

## Introduction

Scleroderma is a rare multisystem autoimmune disorder that is characterized by the presence of a vasculopathy with variable fibrosis of the skin and internal organs. Incidence rates vary from 8 to 56 new cases per million people per year worldwide, whereas the prevalence ranges from 38 to 341 cases per million people [1]. Patients may be categorized based on the degree of skin and associated internal organ involvement. Those with evidence of Raynaud’s phenomenon, thickened skin, puffed-up or swollen fingers, hand stiffness, or fingertip ulcerations should be evaluated for scleroderma. The presence of positive antitopoisomerase I (anti-Scl-70), anticentromere (ACA), and/or anti-RNA polymerase III antibodies, or a positive antinuclear antibody (ANA) with a nucleolar immunofluorescence pattern, further supports the diagnosis [2]. The mainstay of the management of this disease is organ-based symptomatic therapy and consists of an individualized approach to each patient. Nevertheless, patients with severe and diffuse disease will often require systemic immunosuppressive therapy [3]. Prognosis depends mostly on the occurrence of complications, and mortality in patients with scleroderma is increased compared to the general population [4]. Scleroderma renal crisis (SRC), a rare complication of scleroderma, is a life-threatening disorder defined by the presence of hypertension and acute renal failure. Uncertainty surrounds the pathophysiological processes behind the development of SRC. Nonetheless, it has been hypothesized that an essential pathophysiological element in its development is the complement system [5,6]. This system plays a significant role in the protection of the host against microorganisms. Additionally, it acts as a tool for detecting and removing cellular waste and wounded tissue. It is a significant contributor to innate immunity and a component of the humoral immune system’s effector arm. Complement activation increases tissue damage in pathological situations characterized by autoantibodies and immune complexes. A dysfunction in the complement system predisposes to autoimmunity [7,8]. Some patients with SRC have reportedly benefited from eculizumab, a recombinant monoclonal antibody targeted against the complement component C5 [9].

## Case presentation

A 52-year-old female was admitted to Houston Methodist Sugar Land Hospital because of renal dysfunction and hypertension. She has had a five-year history of scleroderma previously confirmed by skin biopsy and positive ANA titers with nucleolar pattern (but negative anti-Scl70 and anticentromere antibodies) and has received regular follow-ups. Her primary symptoms have been Raynaud’s phenomenon and skin changes, and she has been treated with methotrexate 20 mg per os (PO) weekly (qWeek) since her diagnosis. The patient has not been on corticosteroids. Her renal function has been normal throughout her disease course, and the latest reported creatinine value was 0.4 mg/dL in July 2021.

A routine colonoscopy was scheduled in January 2022, but it was canceled because of the patient’s high blood pressure. A blood workup was done later by her rheumatologist, and it showed elevated creatinine. Therefore, she was referred to the Houston Methodist Sugar Land Hospital emergency department (ED). On admission to the hospital, the patient was alert. Her blood pressure was 146/78 mmHg, her pulse rate was 71 beats per minute, her temperature was 97.9°F, her respiratory rate was 20 breaths per minute, and her SpO2 was 99%. The patient was fatigued and had lost 30 lbs in the previous three months. She was also experiencing shortness of breath, chest tightness, vomiting, dizziness, and joint pains. On physical examination, she was noted to have generalized salt and pepper type of hypopigmentation and hyperpigmentation on the face and trunk primarily, with some extension onto the proximal extremities. Hypopigmentation was also noted over the tibias bilaterally. A mild but generalized tapering of the fingertips was present. Diffuse skin thickening was present. No synovitis was detected. The findings of the physical examination of the heart, lungs, abdomen, and nervous system were unremarkable.

Relevant laboratory data on admission is reported in Tables 1-3. Her white blood cell (WBC) count was 3,400/μL (low), red blood cell (RBC) count was 222 × 10^4^/μL (low), hemoglobin level was 6.7 g/dL (low), hematocrit level was 22.2% (low), reticulocyte index was 2.5% (high), and platelet (Plt) count was 49,000/μL (low). Schistocytes were found on peripheral blood smears. Her creatinine (Cr) level was 10.39 mg/dL (high), blood urea nitrogen (BUN) was 132 mg/dL (high), sodium (Na) level was 133 mEq/L (low), chloride (Cl) level was 89 mEq/L (low), potassium (K) level was 3.9 mEq/L, alanine transaminase (ALT) level was 10 IU/L, aspartate transaminase (AST) level was 17 IU/L, alkaline phosphatase (ALP) level was 46 IU/L, total bilirubin level was 0.5 mg/dL, and lactate dehydrogenase (LDH) was 419 IU/L. The urine was positive for protein with 2+ excretion, and the sediment contained 2 red blood cells/high-power field and 2 white blood cells/high-power field, but no granular casts. Moderate bacteriuria was also noted.

**Table 1:** Complete blood count results on admission. WBC: white blood cell, RBC: red blood cell, Hb: hemoglobin, HCT: hematocrit, MCV: mean corpuscular volume, Retic: reticulocyte index, Plt: blood platelet, Fib: fibrinogen

Blood cell count	
WBC	3,400/μL
RBC	222 × 10^4^/μL
Hb	6.7 g/dL
HCT	22.2%
MCV	98.3 fL
Retic	2.5%
Plt	49,000/μL
Fib	188 mg/dL

**Table 2:** Blood chemistry results on admission. Cr: creatinine, BUN: blood urea nitrogen, Na: sodium, Cl: chloride, K: potassium, ALT: alanine transaminase, AST: aspartate transaminase, ALP: alkaline phosphatase, BiliTot: total bilirubin, LDH: lactate dehydrogenase

Blood chemistry	
Cr	10.39 mg/dL
BUN	132 mg/dL
Na	133 mEq/L
Cl	89 mEq/L
K	3.9 mEq/L
ALT	10 IU/L
AST	17 IU/L
ALP	46 IU/L
BiliTot	0.5 mg/dL
LDH	419 IU/L

**Table 3:** Urinalysis results on admission. SG: urine specific gravity, RBC: red blood cell, WBC: white blood cell, HPF: high-power field

Urinalysis	
SG	1.026
pH	6.0
Color	Yellow
Appearance	Cloudy
Protein	2+
RBC	2/HPF
WBC	2/HPF
Cast	(-)
Epithelial	0-10/HPF
Bacteriuria	Moderate

**Figure 1:**
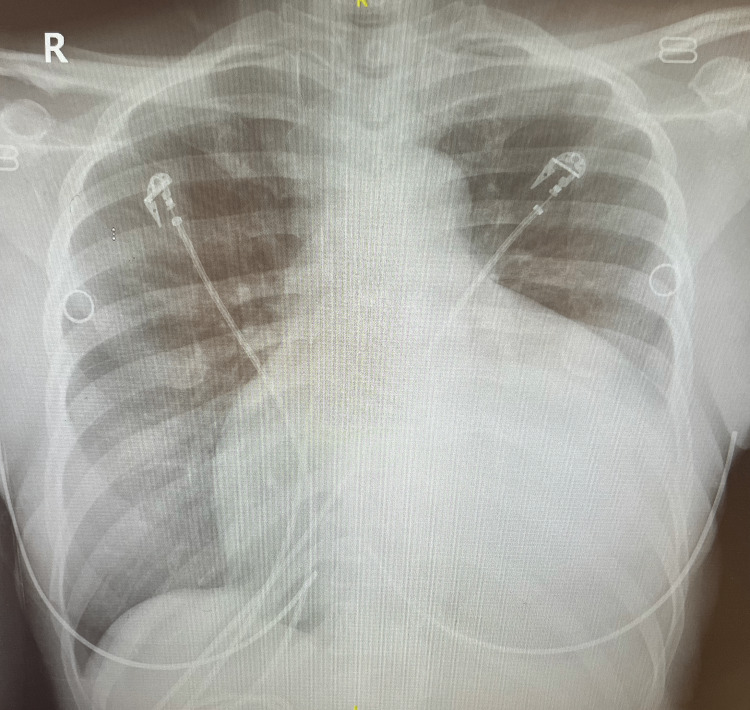
Chest X-ray on admission. Chest X-ray shows significant cardiomegaly with a globular appearance that is compatible with a pericardial effusion.

Chest X-ray showed marked cardiomegaly with a globular configuration most consistent with a pericardial effusion (Figure 1).

**Figure 2:**
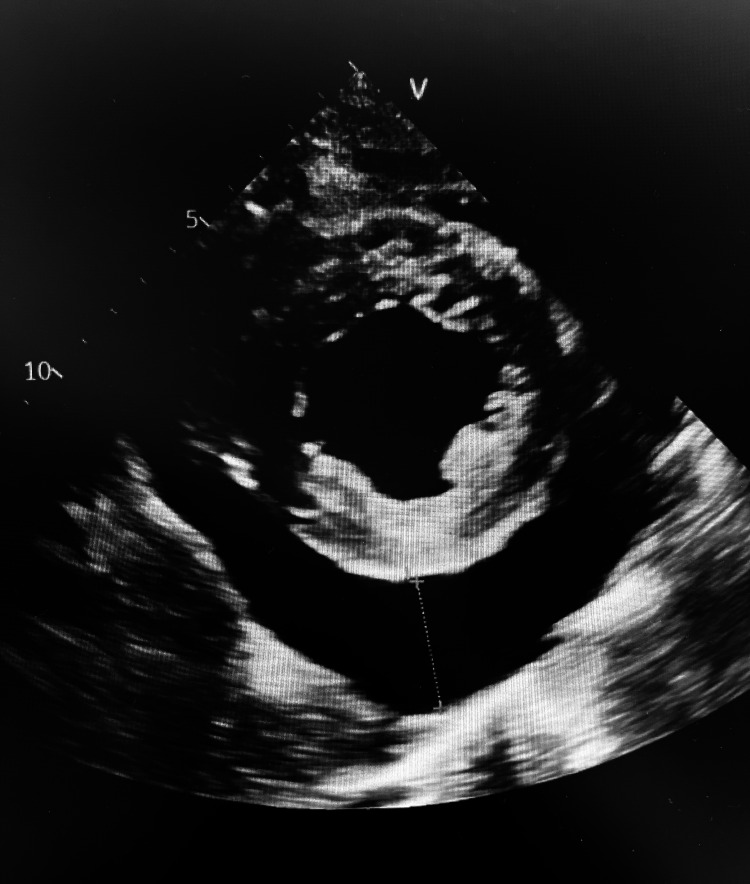
Transthoracic echocardiogram short-axis view showing a large pericardial effusion.

An echocardiogram was performed and confirmed the presence of a large pericardial effusion that was felt to be an early tamponade (Figure 2). The ejection fraction was 40%-45%, and systolic pulmonary artery pressure was 54 mmHg. The patient’s latest echocardiogram performed in August 2020 showed an ejection fraction of 55%-60%, and her pulmonary artery pressure was normal. A small pericardial effusion was noted at that time.

As the patient had hypertension, elevation in serum creatinine with proteinuria, and evidence of microangiopathic hemolytic anemia, she was diagnosed with SRC. The patient was treated with captopril 75 mg PO twice a day (BID), an angiotensin-converting enzyme (ACE) inhibitor that is considered the treatment of choice in SRC. A pericardiocentesis was necessary to aspirate fluid causing early tamponade findings. The patient has not been on corticosteroids, which is appropriate for her current clinical situation since they are contraindicated in SRC [5]. Due to the persistent decline in renal function and decline in clinical status of the patient, a decision was made to administer eculizumab, a complement C5 monoclonal antibody. It was initiated at the initial dose of 900 mg intravenously (IV) qWeek for the first four weeks, followed by 1,200 mg IV qWeek one week later, and then 1,200 mg IV q2Weeks thereafter.

After the treatment with eculizumab was initiated, the hematologic profile of the patient drastically improved, as reported in Table 4. One week later, the patient’s anemia significantly improved with the Hb increasing from 6.7 to 8.5 g/dL, reticulocyte index decreasing from 2.5% to 1%, and LDH decreasing from 419 to 149 IU/L. The patient’s platelet count also normalized, increasing from 49,000 on admission to 270,000/μL. Fibrinogen levels were restored, increasing from 188 to 357 mg/dL. Her renal function showed remarkable improvement, with creatinine decreasing from 10.39 to 4.17 mg/dL.

**Table 4:** Laboratory findings on admission versus after the initiation of eculizumab therapy. Hb: hemoglobin, Retic: reticulocyte index, Plt: blood platelet, Fib: fibrinogen, LDH: lactate dehydrogenase, Cr: creatinine, BUN: blood urea nitrogen

Laboratory findings	On admission	After eculizumab therapy
Hb	6.7 g/dL	8.5 g/dL
Retic	2.5%	1%
Plt	49,000/μL	270,000/μL
Fib	188 mg/dL	357 mg/dL
LDH	419 IU/L	149 IU/L
Cr	10.39 mg/dL	4.17 mg/dL
BUN	132 mg/dL	20 mg/dL

## Discussion

SRC is a rare but severe and life-threatening complication of scleroderma, characterized by hypertension and acute renal failure [6]. One of the major risk factors for developing SRC is the presence of diffuse skin involvement, which was the case in our patient who had generalized skin changes. Another important risk factor, which was not present in our patient, is corticosteroid therapy. A certain immunologic profile also appears to be predictive of the development of SRC. Anti-RNA polymerase III autoantibodies were associated with an increased SRC incidence [5].

The definition of SRC is not generally validated. However, Butler et al. proposed a list of criteria for the diagnosis of this condition (Table 5) [10]. First, hypertension is an important criterion that should be present. Our patient had a systolic blood pressure of 146 mmHg on admission, which is consistent with hypertension. Another criterion is the evidence of renal dysfunction, which is documented by an acute increase in serum creatinine of ≥1.5 times the baseline. On her admission blood workup, the patient had a serum creatinine of 10.39 mg/dL, which is more than 25 times her baseline value of 0.4 mg/dL, reported in July 2021. Since the patient was treated with methotrexate, this drug’s renal toxicity is a differential diagnosis that was considered. Nephrotoxicity is usually seen with high-dose IV methotrexate and is typically non-oliguric and usually reversible [11]. However, this patient’s presentation was more compatible with SRC. In fact, she also had evidence of microangiopathic hemolytic anemia with schistocytes on peripheral blood smears. Target organ dysfunction was also present: the patient complained of chest tightness, and the echocardiogram performed revealed a large pericardial effusion. Therefore, the patient met the criteria necessary for SRC diagnosis.

**Table 5:** Butler et al.’s final core set of items to develop the classification criteria for SRC. SRC: scleroderma renal crisis Source: [10]

Diagnostic criteria for SRC
Blood pressure
Acute increase in blood pressure defined as any of the following: systolic blood pressure ≥ 140 mmHg/diastolic blood pressure ≥ 90 mmHg/an increase in systolic blood pressure of ≥30 mmHg above normal/an increase in diastolic blood pressure of ≥20 mmHg above normal
Kidney injury
Acute kidney injury defined as any of the following: increase in serum creatinine of ≥26.5 μmoles/L (≥0.3 mg/dL) within 48 hours/increase in serum creatinine to ≥1.5 times the baseline, which is known or presumed to have occurred within the prior seven days/urine volume < 0.5 mL/kg/hour for six hours
Microangiopathic hemolytic anemia and thrombocytopenia
New or worsening anemia not due to other causes/schistocytes or other red blood cell fragments on blood smear/thrombocytopenia ≤ 100,000 platelets/mm^3^, confirmed by manual smear/laboratory evidence of hemolysis, including elevated lactate dehydrogenase, reticulocytosis, and/or low or absent haptoglobin/a negative direct antiglobulin test
Target organ dysfunction
Hypertensive retinopathy/hypertensive encephalopathy/acute pericarditis
Renal histopathology
Histopathologic findings on kidney biopsy consistent with SRC

The pathophysiological mechanisms responsible for the development of SRC are not well understood. However, the primum movens is renal endothelial cell damage. This results in intimal proliferation in the renal arteries. The complement system has been thought to be an important pathophysiological factor in the development of SRC [9]. In individuals with scleroderma, the activation of all complement pathways is elevated. Complement markers show a distinctive pattern in patients with SRC, indicating continuous complement consumption [12]. Hypocomplementemia with low C3 and/or C4 levels can sometimes be found in patients with systemic sclerosis and is a predictive factor for poor disease prognosis and vascular damage [13,14]. Furthermore, patients with scleroderma and a poor renal prognosis have C4d deposits in the peritubular capillaries [15]. Finally, C5b-9 deposition was individualized in capillaries of skin biopsies of patients with scleroderma, a finding not observed in healthy patients [16]. Eculizumab is a humanized recombinant immunoglobulin G2/4 monoclonal antibody directed against the complement component C5, thus inhibiting the formation of C5a and C5b-9, limiting endothelial damage [17].

**Figure 3:**
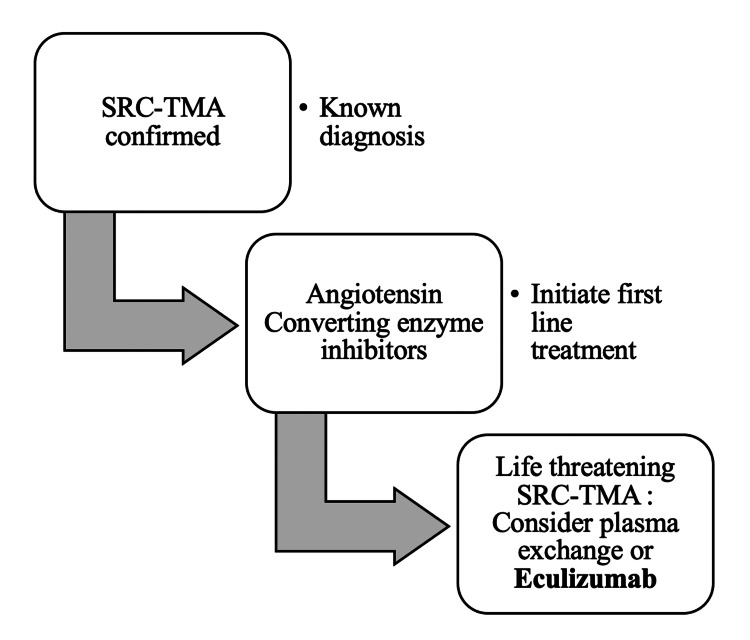
Simplified approach to the management of SRC-TMA. SRC: scleroderma renal crisis, TMA: thrombotic microangiopathy

Upon review of the literature, we found some similar cases of successful improvement of patients diagnosed with SRC upon the initiation of eculizumab. Devresse et al. reported a case of a patient presenting with severe SRC who also presented with extensive thrombotic microangiopathy (TMA) lesions in the kidneys. Evidence of complement activation was found both in serum and in the kidneys with immunofluorescence showing significant deposits of C1q and C4d in the endothelium of renal arterioles. Due to the deteriorating clinical condition with lack of response to early treatment with ACE inhibitors, calcium channel blockers (CCBs), and plasma exchange and in the light of the histological finding, the patient was also treated with eculizumab, which achieved hematologic remission [18]. Likewise, Uriarte et al. described a similar case of a patient diagnosed with SRC and TMA who was treated with eculizumab following a significant decline in hematologic, renal, and cardiac functions. Six months later, the patient achieved full renal recovery with withholding of renal replacement therapy treatment that was no longer needed [19]. On another note, Gouin et al. followed a cohort of 11 SRC-TMA patients, some of whom received eculizumab. The renal outcome remained poor (seven patients had to receive renal replacement therapy at the end of follow-up), including in patients receiving eculizumab. The one-year survival of SRC-TMA patients was 52%, which was similar to historical cohorts [20]. Nonetheless, a proposed algorithm for the management of SRC-TMA, including the use of C5 inhibitors, is shown (Figure 3). When SRC-TMA is confirmed, first-line treatment with ACE inhibitors should be initiated. If refractory or life-threatening, plasma exchange or eculizumab should be considered, although they are not considered as a standard of care yet.

## Conclusions

In conclusion, we report a case of SRC that was unresponsive to initial ACE inhibitor treatment but showed remarkable improvement with the use of eculizumab. Considering the pathophysiologic role of the complement system in SRC and the significant morbidity and mortality of patients with SRC, eculizumab can be a treatment option in patients who are unresponsive to ACE inhibitors. Further studies are needed to assess the potential benefit of early treatment with complement inhibitors in these patients, especially in the setting of life-threatening SRC despite the use of ACE inhibitors.
